# The Influence of Physical Exercise on Head and Neck Structures and Functions Related to Swallowing: A Scoping Review Protocol

**DOI:** 10.1111/1460-6984.70119

**Published:** 2025-08-26

**Authors:** Anittha Mappanasingam, Erin Langman, Rebecca Affoo, Ashwini Namasivayam‐MacDonald

**Affiliations:** ^1^ Faculty of Health Sciences, School of Rehabilitation Science McMaster University Hamilton Ontario Canada; ^2^ University Library, University of Saskatchewan Saskatoon Saskatchewan Canada; ^3^ School of Communication Sciences and Disorders Dalhousie University Halifax Nova Scotia Canada

**Keywords:** deglutition, dysphagia, exercise, physical exercise, swallowing

## Abstract

**Background:**

Dysphagia (swallowing impairment) can lead to malnutrition, dehydration, aspiration pneumonia, social isolation and even death. Current interventions for dysphagia include rehabilitation that improves swallowing physiology and function. Physical exercise is known to directly benefit skeletal muscles and may also benefit non‐targeted skeletal muscles involved in swallowing, as these muscles are often engaged during vigorous exercise. Implementing physical exercise as a dysphagia intervention may help counteract sarcopenic dysphagia and delay symptoms associated with frailty. The objective of this review is to describe current evidence exploring the impact of different forms of physical exercise on structures and functions related to swallowing in both adult humans and animals.

**Methods:**

This study will use PRISMA‐ScR protocol guidelines and JBI methods to ensure proper reporting. Quantitative studies (experimental and quasi‐experimental study designs) and case studies/series that examined either mature animal models or adults who engaged in physical exercise programs. This review will assess any physiological or functional changes to the head and neck structures involved in swallowing assessed by swallowing‐related outcome measures and consider physical exercise programs for the intervention. Studies that include children do not provide details regarding exercises (e.g., dose and type of exercise), or qualitative studies will be excluded. The following databases were searched on 7 April 2024 by an experienced health sciences librarian: MEDLINE, Embase, CINAHL and Scopus. English language articles will be included in the review with no restrictions on publication date. Title and abstract screening will be performed, followed by full‐text screening according to the inclusion criteria. Data regarding type and dose of exercise, along with its effect on the structures involved in swallowing, will be extracted using a proposed data charting tool and presented in tables and figures as appropriate.

**Discussion:**

This scoping review aims to comprehensively describe existing literature examining the relationship between physical exercise and swallowing‐related structures and functions. A detailed synthesis of the current evidence is essential to advance multidisciplinary approaches to dysphagia care, including the integration of physical exercise. Such efforts will enable clinicians to take proactive steps in supporting adults to maintain swallowing function.

**Review Registration Number:**

Open Science Framework: https://osf.io/w98t6/

**WHAT THIS PAPER ADDS?:**

*What is already known on this subject*

- Currently, there are various head‐and‐neck specific exercises that manage and improve dysphagia. While these interventions are effective, they are not easily accessible to patients, making it challenging to integrate them into their daily routines without access to speech‐language pathologists, who are often underfunded. Physical exercise helps maintain targeted muscles; however, evidence on whether these benefits extend to non‐targeted muscles remains limited.

*What this paper adds to existing knowledge*

- This protocol provides a comprehensive synthesis of the existing evidence on the relationship between physical exercise and swallowing‐related structures and functions. The resulting scoping review will inform researchers of key areas for future research.

*What are the potential or actual clinical implications of this work?*

- This scoping review will provide speech‐language pathologists with the existing literature regarding the role of physical exercise on swallowing. This will allow clinicians to adapt current interventions to include interdisciplinary approaches, such as physical exercise, into dysphagia care.

## 1 Background

Dysphagia is the medical term used to describe swallowing impairment due to functional and structural changes to swallowing physiology (Trate et al. 1996). Approximately, 1 in 25 adults will experience dysphagia annually (Bhattacharyya 2014), with older adults more likely to experience difficulties than other age groups (Madhavan et al. 2016; Namasivayam‐MacDonald et al. 2023). The greatest risk of developing dysphagia lies with older adults who present with complex medical conditions, such as neurodegenerative disease, head and neck cancers and neurologic impairments (Madhavan et al. 2016; Namasivayam‐MacDonald et al. 2023; Chen et al. 2022). Another contributor to dysphagia is sarcopenia (the loss of muscle mass and strength), with up to 81% of older adults living with sarcopenic dysphagia (Molfenter 2022; de Sire et al. 2022; Moncayo‐Hernández et al. 2021). As individuals age, the body begins to lose muscle mass, including the tongue, which could result in dysphagia (Dellis et al. 2018; Maeda and Akagi 2016). If mismanaged, dysphagia can lead to malnutrition, aspiration pneumonia and even death (Madhavan et al. 2016; Namasivayam‐MacDonald et al. 2023; Dell'Aquila et al. 2022) and contribute to frailty (Madhavan et al. 2016; Namasivayam‐MacDonald et al. 2023). Dysphagia is also associated with significantly longer hospital stays, leading to higher costs for caregivers and the healthcare system (Patel et al. 2017). Dysphagia also affects an individual's participation in social activities; as they begin having trouble during mealtimes, they may exhibit signs of depression, anxiety and isolation, resulting in decreased quality of life (Namasivayam‐MacDonald et al. 2023; Farri et al. 2007; Bendsen et al. 2022).

### 1.1 Current Interventions for Dysphagia

Currently, clinicians implement both compensatory measures and rehabilitation interventions to manage and improve dysphagia, respectively. These techniques typically involve oropharyngeal structures, such as the tongue, mouth and neck. Compensatory measures involve techniques that help individuals cope with impairments in swallowing, such as postural changes (Easterling 2018; Butler et al. 2013). Rehabilitative interventions involve exercises that improve swallowing physiology and, in turn, function, such as chin tuck against resistance (Inamoto et al. 2018; Huckabee et al. 2023). Current interventions, although useful, are not accessible to all since swallowing‐specific services tend to be limited due to a lack of resources and funding (McGill et al. 2020). As such, healthcare teams would benefit from an understanding of how interdisciplinary approaches may benefit swallowing.

### 1.2 The Role of Physical Exercise for Dysphagia

Physical exercise is defined as planned, repetitive bodily movement performed with the intention to engage in these movements, whereas physical activity is any bodily movement that is unintentional and simply requires energy expenditure (Caspersen et al. 1985). The focus of the current study will be physical exercise involving limb musculature. Physical activity will be excluded, as this may result in minimal change in targeted and non‐targeted skeletal muscles.

Physical exercise impacts breathing by increasing tongue muscle performance, allowing for increased upper airway patency (Kletzien et al. 2016). The swallowing pathway overlaps with the respiratory pathway, and the systems work in coordination to ensure that both swallowing and breathing occur seamlessly (Matsuo and Palmer 2009). Structures, such as the tongue, are involved in both breathing and swallowing. For example, the genioglossus muscle of the tongue helps propel the bolus into the pharynx during swallowing and assists in dilation of the pharynx when breathing (Matsuo and Palmer 2009; McCausland and Anatomy 2023; Fregosi and Ludlow 2014). Interestingly, physical exercise may benefit non‐targeted skeletal muscles, such as the genioglossus (VanRavenhorst‐Bell et al. 2017; VanRavenhorst‐Bell et al. 2018), in addition to targeted muscle groups. Studies have demonstrated that physical exercise increases genioglossus contraction, resulting in stronger tongue musculature (Williams et al. 2000; Vincent et al. 2002). Furthermore, physical exercise may play a role in counteracting sarcopenia, and since sarcopenia is believed to affect head and neck muscles, it is possible that exercise may also benefit these muscles (Beas‐Jiménez et al. 2011).

The potential benefits of exercise on head and neck muscles may vary depending on factors, such as mode, intensity, duration and frequency of the physical exercise (VanRavenhorst‐Bell et al. 2018; Wilson et al. 2012; Karavirta et al. 2011). Although some studies have investigated the impact of exercise modality, such as weightlifting and running, on tongue muscle performance (VanRavenhorst‐Bell et al. 2018), other exercise parameters remain underexplored. Further research is necessary to understand how these factors may influence swallowing.

### 1.3 Rationale for Scoping Review

A preliminary search of PROSPERO, MEDLINE, the Cochrane Database of Systematic Reviews and JBI Evidence Synthesis was conducted on 15 January 2024, and some relevant scoping reviews were identified. A scoping review exploring the effects of whole‐body exercises on swallowing function was previously published; however, it focused on studies involving humans with no restrictions on age (Pu and Yao 2023). Another scoping review explored the benefits of non‐oropharyngeal exercise in the prevention and treatment of dysphagia (Yoshimatsu et al. 2025). Lastly, another review identified non‐traditional approaches to swallowing rehabilitation, with physical exercise identified as one approach (Horyacheva et al. 2023). Our review will delve further into how physical exercise influences structures and functions involved in swallowing. In comparison to other reviews, the current review will have a broader scope, as it will include studies using animal models. Based on a preliminary search, it is evident that there is limited research on this topic. Incorporating animal studies will provide important mechanistic information and serve as a starting point to explore the role of physical exercise on swallowing function in humans. Further, rather than focusing only on the benefits of physical exercise, our focus will be on the overall influence of physical exercise on swallowing. The limited research on the relationship between physical exercise and swallowing requires exploration of both positive and negative effects. When compared with other reviews, our scoping review will have increased specificity regarding the age of study participants and the type of physical activity. Specifically, our review will exclude individuals less than 18 years of age since exercise programs and their impact on physiology typically differ between adults and paediatrics. It will also only highlight evidence resulting from physical exercise interventions and will exclude studies that discuss physical activity only or have combined interventions, while not differentiating between the impact of the exercise intervention.

This scoping review will serve as the basis for future studies looking to explore the influence of exercise in swallowing. Ultimately, by understanding this relationship, swallowing clinicians may consider adapting current approaches to incorporate exercise‐based interventions within interdisciplinary approaches to care (Carnaby and Harenberg 2013). Integrating physical exercise can allow individuals with dysphagia to have accessible ways of managing dysphagia, while they await speech‐language pathology services. This information may also assist in promoting proactive approaches to care, allowing adults to maintain swallowing function by integrating physical exercise into their daily routine.

The primary objective of this scoping review is to describe current evidence exploring the influence of physical exercise on structures and functions involved in swallowing in adult humans (≥18 years). The secondary objective is to investigate the influence of physical exercise on structures and functions involved in swallowing in mature animal models.

## 2 Methods

The proposed scoping review will be conducted in accordance with the JBI methodology for scoping reviews (Peters et al. 2020) and in line with the Preferred Reporting Items for Systematic Reviews and Meta‐Analyses extension for Scoping Review Protocols (PRISMA‐ScR) (Tricco et al. 2018). This scoping review has been registered with Open Science Framework.

### 2.1 Eligibility Criteria

#### 2.1.1 Participants

This review will include studies that included mature animal models considered beyond the range of infancy, or humans 18 years of age or older, regardless of swallowing status. Studies involving children will be excluded as rehabilitation often differs between paediatrics and adults. There will be no restrictions on species or other characteristics of animal models due to the limited studies examining the relationship between physical exercise and swallowing structures and functions.

#### 2.1.2 Concept

The review will only consider physical exercise programs for the intervention. Our operational definition of exercise is planned, repetitive bodily movement performed with the intention to engage in these movements (Caspersen et al. 1985). Therefore, studies that include interventions involving any type of bodily movement that simply requires energy expenditure will be excluded, as the review will focus on purposeful movement. This review will include studies that evaluated participants who engaged in exercise programs using limb musculature, such as aerobic exercise, strength training and balance exercises. Studies will only be included if details of the exercise program, such as mode, frequency and duration of the program, are provided. Studies that categorized participants as physically active or inactive without detailing any exercises will be excluded. Those with combined interventions will be included if the impact of the exercise intervention can be discerned. We will also include studies that measured any physiologic or functional changes to the head and neck structures involved in swallowing. This review will consider studies that include the following swallowing‐related outcome measures: measures of swallowing function, such as videofluroscopic swallowing studies and fibreoptic endoscopic evaluations of swallowing; measures of structures involved in swallowing, such as tongue strength and endurance and biochemical properties of the structures involved in swallowing, such as muscle composition.

#### 2.1.3 Context

This review will consider studies that include participants of all genders, ethnicities and medical diagnoses to maintain a broad scope. This review will not be restricted by geographic location, social setting or healthcare setting.

### 2.2 Information Sources

This scoping review will include quantitative studies (experimental and quasi‐experimental study designs including randomized controlled trials, non‐randomized controlled trials, before and after studies, and interrupted time‐series studies) and case studies/series. Conference abstracts, opinion papers, review papers and qualitative studies will be excluded.

The databases to be searched include MEDLINE (Ovid), Embase (Ovid), CINAHL (EBSCO) and Scopus (Elsevier). Sources of unpublished studies and grey literature to be searched include the WHO International Clinical Trials Registry Platform, ClinicalTrials.gov and ProQuest Dissertation and Thesis. Articles published in English, or previously translated into English, will be considered, as the authors do not have access to translation services. There is no restriction on the publication date.

### 2.3 Search Strategy

The search strategy will aim to locate both published and unpublished primary studies, and reviews. An initial limited search of MEDLINE was undertaken to identify articles on the topic. The text words contained in the titles and abstracts of relevant articles, and the index terms used to describe the articles, were used by a health sciences librarian to develop a full search strategy for MEDLINE (Ovid) (see Appendix I). Text words and index terms were identified, in part, using WordFreq, the word frequency tool in Systematic Review Accelerator (Institute for Evidence‐Based Healthcare and Bond University) (SR‐Accelerator), and Yale MeSH Analyzer (Cushing/Whitney Medical Library, Yale University) (Yale MeSH Analyzer). The search strategy for MEDLINE was peer‐reviewed by a second health sciences librarian using Peer Review of Electronic Search Strategies (PRESS) (McGowan et al. 2016). The search strategy, including all identified keywords and index terms, will be adapted for each included information source. The reference lists of articles included in the review will be screened for additional papers.

### 2.4 Selection Process

Following the search, all identified records will be collated and uploaded into Covidence, and duplicates will be removed. All records for all phases of the review will be completed and stored using Covidence data extraction software. Following a pilot test, titles and abstracts will then be screened by four independent reviewers for assessment against the inclusion criteria. Full texts of selected citations will be assessed in detail against the inclusion criteria by two independent reviewers. Reasons for exclusion of full‐text papers that do not meet the inclusion criteria will be recorded and reported in the scoping review. Any disagreements that arise between the reviewers at each stage of the selection process will be resolved through discussion with a third reviewer.

### 2.5 Data Collection

Data will be extracted from papers included in the scoping review by two independent reviewers using a data charting tool developed by the reviewers. The data extracted will include specific details about the population, concept, context, methods and key findings relevant to the review question. A data charting tool is provided (see Appendix II). The tool will be modified and revised as necessary during data extraction. Modifications will be detailed in the full scoping review. Any disagreements that arise between the reviewers will be resolved through discussion or with a third reviewer. Authors of papers will be contacted to request missing or additional data, where required. A maximum of two attempts to contact the corresponding author, and/or co‐authors, where information is available, will be made within a 1‐month timeframe.

### 2.6 Data Items/Synthesis

Data collected from the data charting tool (Appendix II) will be presented in tables and graphs as appropriate. The results of the search will be reported in full in the final scoping review and presented in a PRISMA flow diagram (Page et al. 2021). Descriptive analysis techniques will be used to synthesize, interpret and report quantitative data. This will involve commonly used interventions, describing patterns in the findings and highlighting gaps across the studies.

## 3 Discussion

The proposed scoping review aims to describe existing evidence on the influence of physical exercise on swallowing‐related structures and functions in adult humans and animals. It will identify knowledge gaps within the current literature to guide future research and inform potential advancements in clinical practice, rehabilitation programs and policy changes aimed at enhancing access to care for individuals at risk of dysphagia. Understanding the relationship between physical exercise and swallowing can support interdisciplinary collaboration among swallowing clinicians and other healthcare professionals, such as physiotherapists, personal support workers and family physicians. Such interdisciplinary efforts could help implement more patient‐familiar management strategies, such as physical exercise, to proactively maintain swallowing function and improve patient outcomes, particularly in populations vulnerable to dysphagia and with limited access to current dysphagia care.

## Conflicts of Interest

The authors declare no conflicts of interest.

## Data Availability

Data sharing is not applicable to this article as no new data were created or analysed in this study.

## Appendix I Search Strategy

### I.1

MEDLINE ALL 1946 to 5 April 2024

Search conducted on 7 April 2024.

Lines 1–12 adapted from Yin, D., Engracia, M. V., Edema, M. K., Clarke, D. C. (preprint). A PubMed search filter for efficiently retrieving exercise training studies. *sportRxiv*. https://doi.org/10.51224/SRXIV.353.

**Table:**

#	Searches	Results
1	Physical conditioning, human/	2916
2	Physical Conditioning, animal/	15 783
3	Circuit‐based exercise/	116
4	Endurance training/	618
5	High‐intensity interval training/	2327
6	Plyometric exercise/	842
7	Resistance training/	12 839
8	Exercise therapy/	50 755
9	Aquatic therapy/	29
10	Exercise movement techniques/	884
11	Exercise/ and training.ti, ab, kf.	24 251
12	(interval training or HIIT or cardio training or cardiovascular training or training program* or exercise program* or resistance training or circuit training or concurrent training or aerobic training or anaerobic training or endurance training or sprint interval or aquatic training or exercise training or fitness training or functional training or weight lifting or weightlifting or weight training or bodyweight training or body weight training or strength training or swim training or ((game‐based or exergam* or exer‐gam* or fitness gam*) and training)).ti, ab, kf.	115 092
13	or/1–12	175 581
14	Deglutition/	11 905
15	Deglutition Disorders/	24 521
16	Tongue/	20 795
17	Masticatory muscles/	8078
18	Masseter muscle/	4981
19	Pterygoid muscles/	1019
20	Temporal muscle/	2710
21	Neck muscles/	6496
22	Pharyngeal muscles/	1739
23	Velopharyngeal Sphincter/	84
24	Pharynx/	20 497
25	Esophagus/	45 002
26	Esophageal sphincter, upper/	564
27	Esophagogastric junction/	8807
28	Esophageal sphincter, lower/	1886
29	Velopharyngeal sphincter/	84
30	Laryngeal muscles/	2536
31	Palatal muscles/	647
32	dysphagia.ti, ab, kf.	36 556
33	((swallow* or deglutit*) adj3 (disorder* or impair* or dysfunction* or disfunction* or difficult*)).ti, ab, kf.	10 193
34	((structur* or muscle* or musculature or pathway* or physiolog* or function*) adj3 (head or neck or swallow* or masticat* or tongue or deglutit*)).ti, ab, kf.	27 604
35	(buccinator or cricopharyngeus or esophag* or genioglossus or geniohyoid or hyoglossus or infrahyoid or laryngeal or larynx or lateral cricoarytenoid or lateral pterygoid or levator veli palatini or masseter or medial constrictor or medial pterygoid or musculus uvulae or mylohyoid or orbicularis oris or oropharyngeal or oropharynx or palatoglossus or palatopharyngeal or palatopharyngeus or pharyngeal or pharynx or pterygoid or salpingopharyngeus or styloglossus or stylopharyngeus or superior constrictor or suprahyoid or temporalis or tensor veli palatini or thyroarytenoid or thyropharyngeus or transverse arytenoid or velopharyngeal or velopharynx or verticalis).ti, ab, kw.	309 840

## Appendix II Data Extraction Instrument

### II.1

**Table:**

Publication details	Study title
	First Author
	Year of Publication
	Country
Study details	Objective
	Inclusion Criteria
	Exclusion Criteria
Methodological information	Type of Study
	Intervention Components (type, materials used)
	Intervention Dose (frequency, duration)
	Session Duration
	Comparator
	Outcome Measure(s)
Participant information	Population (animals or humans)
	Age
	Gender
	Sample Size
Findings/Discussion	Results
	Author's Conclusion
	Limitations
	Future Directions
